# Anticipated correlation between lean body mass to visceral fat mass ratio and insulin resistance: NHANES 2011-2018

**DOI:** 10.3389/fendo.2023.1232896

**Published:** 2023-09-13

**Authors:** Ya Shao, Longti Li, Huiqin Zhong, Xiaojun Wang, Yu Hua, Xu Zhou

**Affiliations:** ^1^ Department of Health Management Center, TaiHe Hospital, Hubei University of Medicine, Shiyan, China; ^2^ Department of Nursing, TaiHe Hospital, Hubei University of Medicine, Shiyan, China; ^3^ Department of Gastroenterology, TaiHe Hospital, Hubei University of Medicine, Shiyan, China

**Keywords:** insulin resistance, lean body mass, NHANES, visceral fat mass, HOMA-IR

## Abstract

**Objective:**

The relationship between body composition and insulin resistance (IR) is controversial. This study aimed to thoroughly examine the correlation between adipose tissue, lean body mass, and IR as evaluated by the Homeostatic Model Assessment (HOMA-IR).

**Methods:**

In this cross-sectional study, we utilized data from the National Health and Nutrition Examination Survey (NHANES) conducted between 2011 and 2018. Our study included 4981 subjects, and we employed multiple linear regression, smoothed curve fitting, threshold, and saturation effect analysis to investigate the relationship between lean body mass, visceral fat mass, and IR. Also, we used the lean body mass to visceral fat ratio (Log LM/VFM) as a proxy variable to analyze its association with IR alone.

**Results:**

The study discovered a negative link between lean body mass and IR, but the visceral fat mass was positively correlated after correcting for covariates. A negative correlation was observed when the alternative variable Log LM/VFM was analyzed separately for its association with IR. This association was present regardless of whether the exposure variables were analyzed as continuous or categorical. The data analysis revealed a nonlinear relationship between Log LM/VFM and IR, as evidenced by the generalized additive model. In addition, a threshold effect with a critical value of 1.80 and a saturation effect with a critical point of 2.5 were also observed. Further subgroup analysis for sex, age, BMI, active levels, hypertension, and diabetes showed considerable robustness between the relationship of Log LM/VFM and IR.

**Conclusion:**

Maintaining a proper ratio of lean body mass and visceral fat is beneficial for decreasing IR.

## 1 Introduction

Diabetes has become a significant threat to human health, continuously challenging healthcare systems worldwide (1). The prevalence of diabetes is increasing due to factors such as rapid socio-economic development, deepening urbanization, and the adoption of unhealthy lifestyles, including poor diets and sedentary behaviors (2, 3). According to the International Diabetes Federation (4), a staggering 537 million people worldwide already have diabetes, and by 2045 years that number will grow to 783 million. The prevalence among adults aged 20-79 is 10.5% and will reach 12.2% in 2045. Diabetes is a chronic, lifelong disease that causes ongoing damage to multiple organs and systems in the body, leading to various complications and an overall disease burden. Besides the conventional risk of cardiovascular disease, diabetes is also associated with an increased risk of death from cancer, dementia, and various infections (5).

In the development of diabetes, Insulin resistance (IR) is one of the key pathophysiologic players (6). IR is a pathological condition that impairs insulin-induced glucose uptake and utilization in insulin-sensitive tissues (7). IR states result in the inhibition of adipose tissue catabolism, impaired glucose uptake in muscle, and inhibition of gluconeogenesis in the liver, ultimately leading to various metabolic diseases (8). Numerous studies have established a strong correlation between IR and the development or adverse outcomes of metabolic disorders, including coronary artery disease (9), heart failure (10), cardiovascular disease (11), diabetes (12), and hypertension (13).

Researchers have conducted extensive studies regarding the influencing factors related to IR, of which obesity is a vital link (14, 15). Obesity can lead to chronic inflammation of multiple organs and tissues throughout the body, increasing the risk of many diseases, especially IR (16). When evaluating obesity, the most commonly used indicator is body mass index (BMI). However, it does not provide a valid assessment of the distribution of muscle and fat throughout the body, making it difficult to assess the health risks associated with obesity comprehensively (17). In particular, the mortality rate observed in overweight and obese people with cardiovascular disease was lower than in normal-weight patients, known as the obesity paradox (18).

New epidemiologic studies have shown that precisely measured body composition correlates more strongly with cardiovascular disease than BMI. Researchers then used the ratio of fat to muscle to assess the distribution of muscle and fat. It can be used as a marker of sarcopenic obesity to assess cardiometabolic risk (19). In a study conducted by Yu et al., it was found that the fat-to-muscle ratio had a J-shaped correlation with all-cause mortality within the UK Biobank cohort population (20). Seo et al. discovered a significant correlation between the fat-to-muscle ratio, metabolic syndrome, and IR (21). However, recent studies have shown that not all fat in the body is harmful. Subcutaneous and lower body (buttock and leg) adipose tissue may be protective (22, 23), while visceral fat accumulation may pose significant health problems (24). Thus, the fat-to-muscle ratio marker may ignore health risks that differ between different fats in human tissues. Therefore, we used a novel index, lean body mass to visceral fat mass ratio, to assess its correlation with IR.

## 2 Materials and methods

### 2.1 Study design and participants

**Figure 1:**
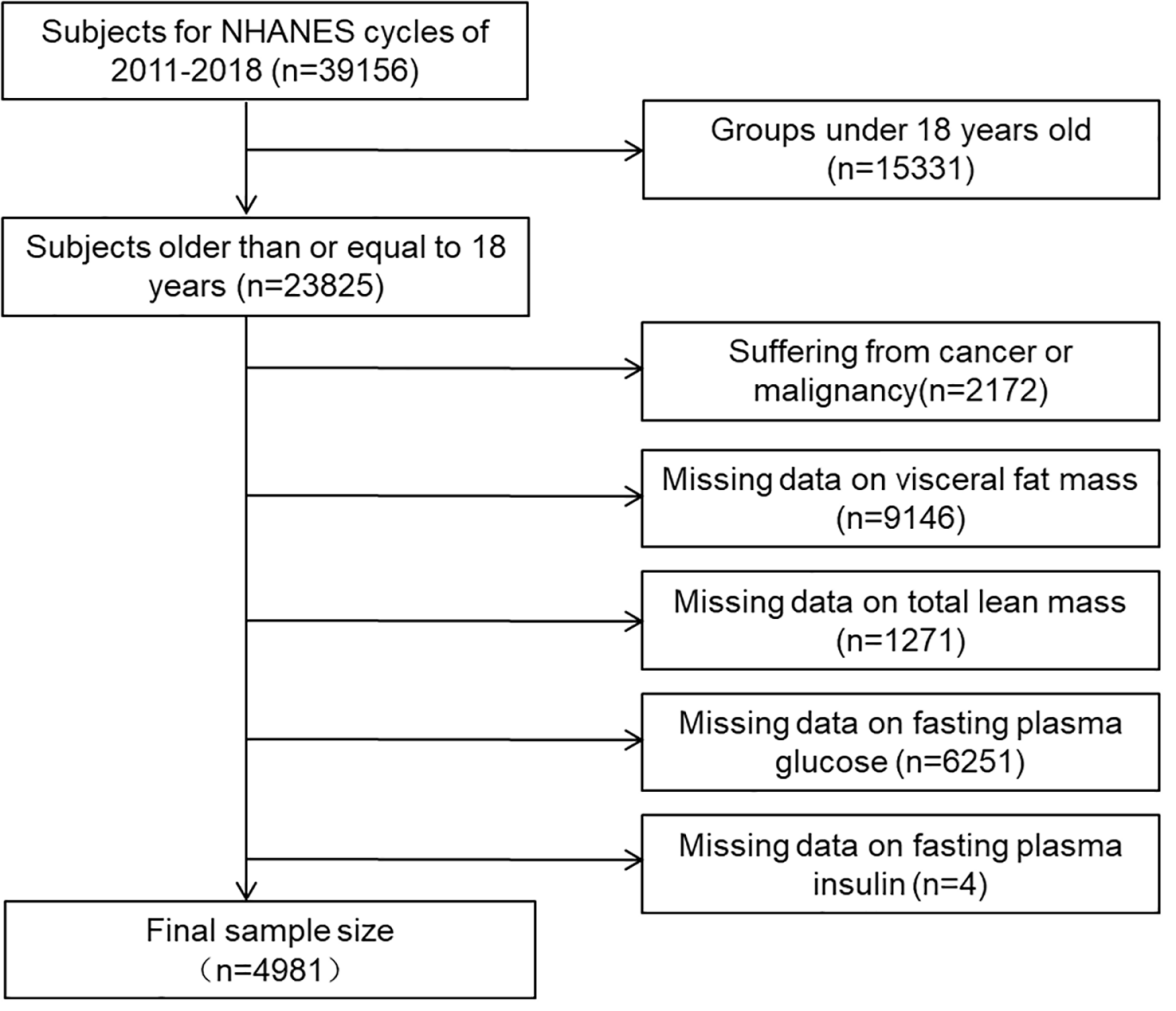
Flow chart for the selection of subjects.

This cross-sectional study used data from the National Health and Nutrition Examination Survey (NHANES), a research program investigating people’s nutrition and health status in the United States. The survey has been ongoing since the 1960s and involves standardized surveys, physical examinations, laboratory tests, and other methods to gather health-related indicators from the population. The survey was conducted on a 2-year cycle using a multistage probability sampling design for the U.S. sample. Within each survey cycle, approximately 30 counties were selected for interviews from the approximately 3,000 counties in the United States. This study utilized data from 2011 to 2018, as only these cycles collected information on visceral fat mass. The National Center for Health Statistics Institutional Review Board of the United States approved the survey, and informed consent was obtained from all NHANES participants before implementation. People older than 18 years who have completed dual-energy X-ray absorptiometry will be included in this study. Individuals with tumors, lacking visceral fat and lean body mass information, and those unable to calculate HOMA-IR variables (fasting blood glucose and plasma insulin) were excluded. The final sample size included 4981 study subjects, 2542 men and 2349 women, and an average age of 37.58 ± 12.30 years. The selection process is illustrated in **Figure 1**.

### 2.2 Exposure and outcome variables

This study used the lean body mass to visceral adiposity mass ratio as an exposure variable. Dual-energy X-ray was employed to determine these variables, a criteria method for assessing body composition and providing insight into the distribution of muscle and fat throughout the body. The Hologic APEX version 4.0 software was used to analyze the data from the whole-body scan. Before the examination began, the subject was required to remove the metal objects from his body. Exclusions were made for pregnant individuals, those using radiology contrast agents within seven days, and those who weighed more than 450 pounds or were taller than 6 feet 5 inches. All examinations were completed by trained and certified radiologic technologists, and each participant’s scan information was reviewed and analyzed by an independent quality control center of the NHANES (25). Body composition, anthropometric measurements, and blood specimen collection were completed at the mobile examination center, which took 40 minutes to 4 hours per subject.

In this study, the outcome variable was IR, and the participants’ IR status was assessed using the Homeostatic Model Assessment for IR (HOMA-IR), a method widely used for quantifying IR. This method involves using the following formula to calculate HOMA-IR: fasting glucose (mg/dL) multiplied by fasting insulin (μU/mL) divided by 405 (26). These fasting tests require a 9-hour fast or more, and participants have blood specimens collected in a mobile screening vehicle. Blood specimens were transported to an independent testing laboratory for testing. Detailed procedures for collecting and testing blood specimens were described in the NHANES Laboratory Operations Manual (27).

### 2.3 Covariates

We used general demographic information on participants, including sex, age, race, marital status, educational attainment, poverty income ratio (PIR), anthropometric measurements (BMI, waist circumference), and lifestyle status, including activity, smoking, and alcohol consumption. Trained health technicians measured anthropometric data at mobile screening centers following standard procedures (28).

BMI was calculated as weight in kilograms divided by the square of height in meters. Waist circumference was measured bilaterally using a tape measure at the level of the superior iliac crest. Physical activity was evaluated using a standardized questionnaire. Participants were asked to report the number of days they engaged in various activities in the previous week and the average number of minutes per day spent on these activities. According to the relevant guidelines (29), physical activity levels were categorized as active, inactive, and completely inactive. “Active” was defined as >75 minutes of vigorous or >150 minutes of moderate-intensity weekly exercise. “Inactivity” includes physical activity that does not meet the above criteria. “Completely inactive” refers to a physically inactive person. Smoking was categorized into three categories: never, former, and current. Never-smokers were individuals who had smoked no more than 100 cigarettes in their lifetime. The former were those who had smoked 100 or more in their lifetime but were currently abstaining from smoking. On the other hand, current smokers were defined as those who had smoked 100 or more in their lifetime and were currently smoking. Alcohol consumption was determined by a cut-off of >12 drinks per year, and no alcohol consumption was defined as consuming less than 12 drinks per year.

In addition, we examined the impact of underlying chronic diseases such as hypertension and diabetes. Hypertension was described as previously diagnosed hypertension, use of antihypertensive medication, or non-same day, systolic blood pressure ≥140 mm Hg or diastolic blood pressure ≥90 mm Hg measured on three or more occasions. Diabetes was defined as a previous diagnosis, glucose-lowering medication or insulin use, fasting glucose ≥126 mg/dL, 2-hour OGTT ≥200 mg/dL, or HbA1c ≥6.5%. In addition, we considered the effects of other covariates, including total cholesterol, triglycerides, alanine aminotransferase (ALT), and blood uric acid.

### 2.4 Statistical analysis

Continuous variables were reported using the mean and standard deviation or the median and interquartile range. Categorical variables were presented as the number of cases and percentages. To compare differences in quartiles based on HOMA-IR, analysis of variance was used for continuous data, and the chi-square test was used for categorical variables. Mobile Examination Centre (MEC) test weights were employed in the analysis to improve data representativeness. Linear regression was utilized to examine the correlation between exposure and outcome variables. As the ratio of lean body mass to visceral fat mass and the HOMA-IR did not follow a Gaussian distribution, Log 10 transformations were employed, and the alternative variables Log LM/VFM, and Log HMOA-IR were used, respectively.

In the regression analysis, three models were considered. These were crude model, which did not adjust for any confounders; model 1, which adjusted for sex, age, and race; and model 2, which further accounted for marital status, education, PIR, BMI, waist circumference, activity, smoking, alcohol consumption, hypertension, diabetes, total cholesterol, triglycerides, ALT, and blood uric acid based on model 1. A generalized additive model investigated the correlation between Log LM/VFM and Log HMOA-IR. In contrast, a piece-wise linear regression model was employed to analyze the threshold effect of Log LM/VFM on HOMA-IR. The study also included stratification by sex, age, BMI, activity, hypertension, and diabetes to examine their potential impact on the outcomes. The statistical analyses were performed using EmpowerStats (www.empowerstats.com) and R software (http://www.r-project.org).

## 3 Results

**Table 1** displays the comprehensive characteristics of the research population. The participants were categorized into four groups based on the HOMA-IR quartiles. The comparison among the groups indicated noteworthy differences in age, race, marital status, education, PIR, alcohol consumption, hypertension, diabetes, waist circumference, BMI, cholesterol, triglyceride, ALT, blood uric acid, lean body, and visceral fat mass.

**Table 1:** Characteristics of participants.

Characteristic	HOMA-IR				
	Q1	Q2	Q3	Q4	P-value
	<1.4	1.40-2.27	2.28-3.94	≥3.95	
N	1245	1245	1245	1246	
Sex, n (%)					0.561
Male	619 (49.72)	628 (50.44)	643 (51.65)	652 (52.33)	
Female	626 (50.28)	617 (49.56)	602 (48.35)	594 (47.67)	
Age (mean ± SD, year)	36.43 ± 11.94	36.46 ± 12.23	37.77 ± 12.69	39.57 ± 12.10	<0.001
Race, n (%)					<0.001
Mexican American	112 (9.00)	171 (13.73)	228 (18.31)	261 (20.95)	
Non-Hispanic White	488 (39.20)	443 (35.58)	389 (31.24)	392 (31.46)	
Non-Hispanic Black	237 (19.04)	242 (19.44)	251 (20.16)	271 (21.75)	
Other Race	408 (32.77)	389 (31.24)	377 (30.28)	322 (25.84)	
Marital status, n (%)					0.007
Married/cohabiting	675 (54.22)	653 (52.45)	689 (55.34)	712 (57.14)	
Widowed/divorced/separated	146 (11.73)	156 (12.53)	168 (13.49)	161 (12.92)	
Never married	338 (27.15)	323 (25.94)	273 (21.93)	293 (23.52)	
Unclear	86 (6.91)	113 (9.08)	115 (9.24)	80 (6.42)	
Education, n (%)					<0.001
Under high school	179 (14.38)	202 (16.22)	228 (18.31)	268 (21.51)	
High school or equivalent	236 (18.96)	241 (19.36)	246 (19.76)	247 (19.82)	
Above high school	744 (59.76)	688 (55.26)	656 (52.69)	651 (52.25)	
Unclear	86 (6.91)	114 (9.16)	115 (9.24)	80 (6.42)	
PIR, n (%)					0.004
≤1.26	356 (28.59)	381 (30.60)	377 (30.28)	408 (32.74)	
1.27-3.17	371 (29.80)	350 (28.11)	394 (31.65)	404 (32.42)	
≥3.18	421 (33.82)	412 (33.09)	364 (29.24)	327 (26.24)	
Unclear	97 (7.79)	102 (8.19)	110 (8.84)	107 (8.59)	
Activity, n (%)					0.175
Active	668 (53.65)	676 (54.30)	680 (54.62)	676 (54.25)	
Less active	85 (6.83)	86 (6.91)	97 (7.79)	88 (7.06)	
Inactive	487 (39.12)	483 (38.80)	468 (37.59)	476 (38.20)	
Smoking, n (%)					0.113
Never	373 (29.96)	414 (33.25)	378 (30.36)	377 (30.26)	
Former	88 (7.07)	92 (7.39)	112 (9.00)	116 (9.31)	
Current	139 (11.16)	117 (9.40)	135 (10.84)	150 (12.04)	
Unclear	645 (51.81)	622 (49.96)	620 (49.80)	603 (48.39)	
Alcohol, n (%)					0.001
Yes	886 (71.16)	866 (69.56)	847 (68.03)	787 (63.16)	
No	19 (1.53)	23 (1.85)	19 (1.53)	28 (2.25)	
Unclear	340 (27.31)	356 (28.59)	379 (30.44)	431 (34.59)	
Hypertension, n (%)					<0.001
Yes	196 (15.74)	219 (17.59)	336 (26.99)	475 (38.12)	
No	1049 (84.26)	1026 (82.41)	909 (73.01)	771 (61.88)	
Diabetes, n (%)					<0.001
Yes	30 (2.41)	49 (3.94)	111 (8.92)	395 (31.70)	
No	1215 (97.59)	1196 (96.06)	1134 (91.08)	851 (68.30)	
Waist Circumference (mean ± SD, cm)	84.09 ± 10.76	90.57 ± 12.34	98.13 ± 13.18	109.70 ± 15.56	<0.001
BMI (mean ± SD, kg/m2)	23.78 ± 4.34	26.37 ± 4.95	29.33 ± 5.47	33.90 ± 6.94	<0.001
Total Cholesterol (mean ± SD, mg/dL)	180.82 ± 37.18	185.36 ± 37.97	190.76 ± 41.36	193.44 ± 42.59	<0.001
Triglyceride (median (IQR), mg/dL)	66.00 (47.00-94.00)	81.00 (57.00-115.52)	101.00 (69.00-147.00)	129.00 (91.00-193.00)	<0.001
ALT (median (IQR),U/L)	17.00 (14.00-23.00)	19.00 (15.00-25.00)	21.00 (16.00-29.00)	26.00 (19.00-39.00)	<0.001
Uric acid (mean ± SD, mg/dL)	4.96 ± 1.25	5.15 ± 1.26	5.51 ± 1.37	5.81 ± 1.47	<0.001
Lean body mass (mean ± SD, kg)	48.74 ± 11.03	51.24 ± 11.81	54.43 ± 12.04	60.73 ± 13.31	<0.001
Visceral fat mass (median (IQR),kg)	0.26 (0.19-0.37)	0.36 (0.24-0.51)	0.49 (0.34-0.66)	0.65 (0.49-0.85)	<0.001
Log LM/VFM	2.27 ± 0.21	2.16 ± 0.22	2.06 ± 0.21	1.98 ± 0.19	<0.001

HOMA, homeostasis model assessment for insulin resistance; SD, standard deviation; IQR, interquartile range; PIR, ratio of family income to poverty; BMI, body mass index; ALT, alanine aminotransferase; LM/VFM, total lean mass/visceral fat mass.

### 3.1 Association of lean body mass to visceral fat mass ratio with IR

The results from the regression analysis are presented in **Table 2**. The results indicate that after adjusting for covariates, total lean body mass exhibited a negative association with HOMA-IR, both in its continuous (β =-0.03; 95% CI: -0.05, -0.02) and converted as a categorical variable. Specifically, the highest quartile of total lean mass was related to lower HOMA-IR (β=-0.10; 95% CI: -0.14, -0.06). When analyzed as a continuous variable, the study found a positive association between visceral fat mass and HOMA-IR (β = 0.08; 95% CI: 0.07, 0.09). Additionally, when visceral fat mass was considered a categorical variable, the highest quartile of visceral fat mass was associated with a higher level of HOMA-IR (β = 0.20; 95% CI: 0.17, 0.24).

**Table 2:** Association of total lean mass, visceral fat and Log LM/VFM with HOMA-IR.

Independent variables	Crude Model		Model I		Model II	
	β (95%CI)	P-value	β (95%CI)	P-value	β (95%CI)	P-value
Total lean mass						
Per-SD increase	0.13 (0.12, 0.14)	<0.001	0.23 (0.22, 0.24)	<0.001	-0.03 (-0.05, -0.02)	<0.001
Q1	Reference		Reference		Reference	
Q2	0.09 (0.06, 0.12)	<0.001	0.17 (0.14, 0.20)	<0.001	-0.06 (-0.08, -0.03)	<0.001
Q3	0.15 (0.12, 0.18)	<0.001	0.32 (0.29, 0.35)	<0.001	-0.08 (-0.11, -0.05)	<0.001
Q4	0.31 (0.28, 0.33)	<0.001	0.53 (0.49, 0.56)	<0.001	-0.10 (-0.14, -0.06)	<0.001
*P* for trend	<0.001		<0.001		<0.001	
Visceral fat mass						
Per-SD increase	0.20 (0.19, 0.20)	<0.001	0.23 (0.22, 0.24)	<0.001	0.08 (0.07, 0.09)	<0.001
Q1	Reference		Reference		Reference	
Q2	0.15 (0.12, 0.17)	<0.001	0.18 (0.16, 0.20)	<0.001	0.06 (0.04, 0.09)	<0.001
Q3	0.32 (0.29, 0.34)	<0.001	0.37 (0.35, 0.40)	<0.001	0.14 (0.11, 0.17)	<0.001
Q4	0.51 (0.49, 0.53)	<0.001	0.60 (0.58, 0.63)	<0.001	0.20 (0.17, 0.24)	<0.001
*P* for trend	<0.001		<0.001		<0.001	
Log LM/VFM						
Per-SD increase	-0.17 (-0.18, -0.16)	<0.001	-0.21 (-0.22, -0.20)	<0.001	-0.07 (-0.08, -0.06)	<0.001
Q1	Reference		Reference		Reference	
Q2	-0.14 (-0.16, -0.11)	<0.001	-0.19 (-0.22, -0.17)	<0.001	-0.05 (-0.07, -0.03)	<0.001
Q3	-0.28 (-0.31, -0.26)	<0.001	-0.37 (-0.40, -0.34)	<0.001	-0.12 (-0.14, -0.09)	<0.001
Q4	-0.44 (-0.46, -0.41)	<0.001	-0.55 (-0.58, -0.53)	<0.001	-0.17 (-0.20, -0.14)	<0.001
*P* for trend	<0.001		<0.001		<0.001	

HOMA, homeostasis model assessment for insulin resistance; β, partial regression coefficient; CI, confidence interval; LM/VFM, Lean mass/Visceral fat mass.

Crude Model: no covariates were adjusted. Model 1: sex, age and race were adjusted. Model 2: sex, age, marital status, race, education, family income to poverty ratio, BMI, waist circumference, activity, smoking, alcohol, hypertension, diabetes, total cholesterol, triglyceride, ALT, and blood uric acid.

The study found a significant inverse relationship between HOMA-IR and Log LM/VFM. This association was observed both as a continuous variable (β=-0.07; 95% CI: -0.08, -0.06) and in categorical variables. Furthermore, individuals in the upper quartile of Log LM/VFM were found to have relatively lower HOMA-IR (β = -0.17; 95% CI: -0.20, -0.14).

### 3.2 Smoothing curve fitting and threshold effect analysis

**Figure 2:**
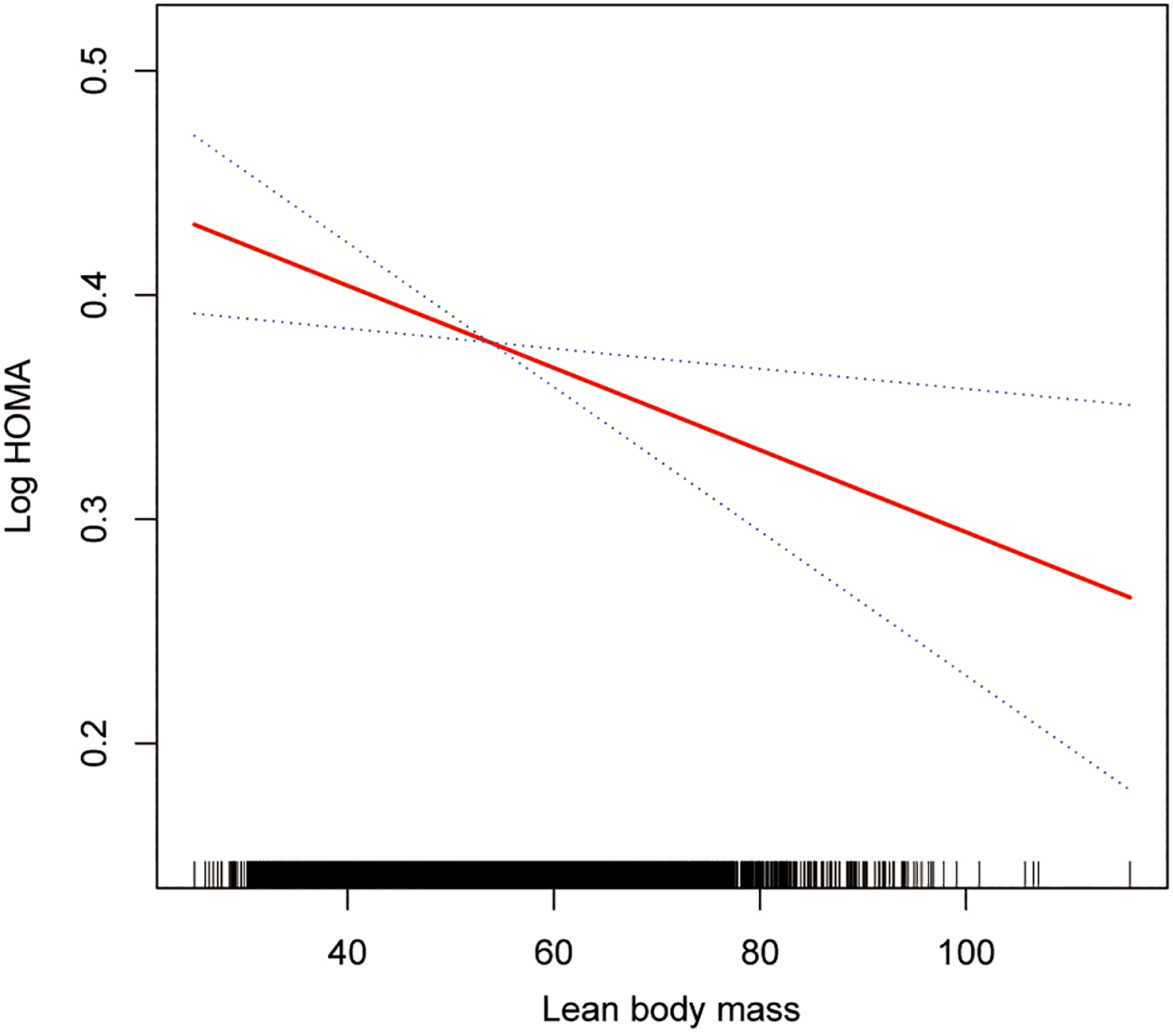
Association between lean body mass and IR. The solid rad line represents the smooth curve fit between variables. Blue bands represent the 95% of confidence interval from the fit. All adjusted covariates were the same as in the regression analysis.

**Figure 3:**
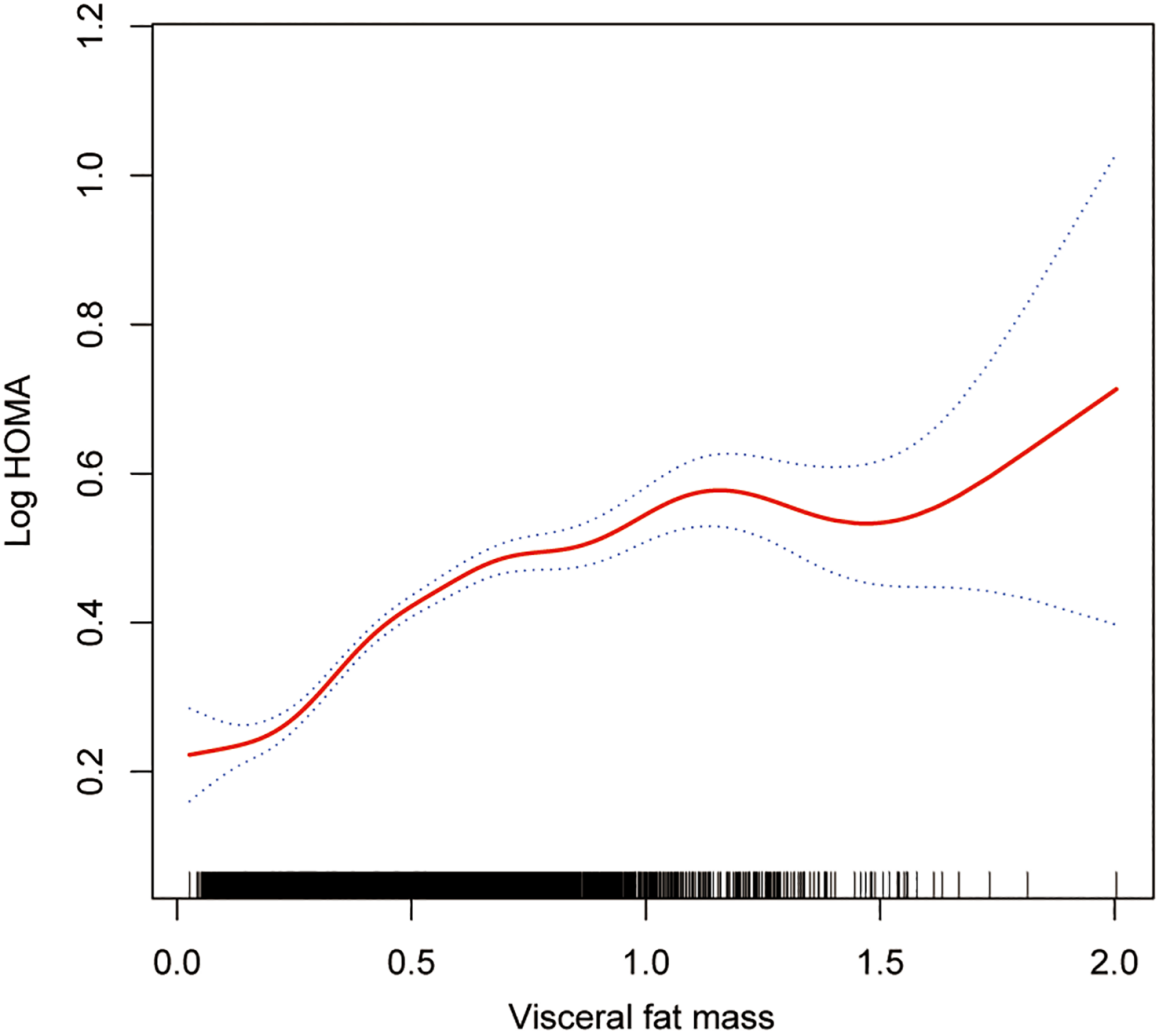
Association between visceral fat mass and IR. The solid rad line represents the smooth curve fit between variables. Blue bands represent the 95% of confidence interval from the fit. All adjusted covariates were the same as in the regression analysis.

**Figure 4:**
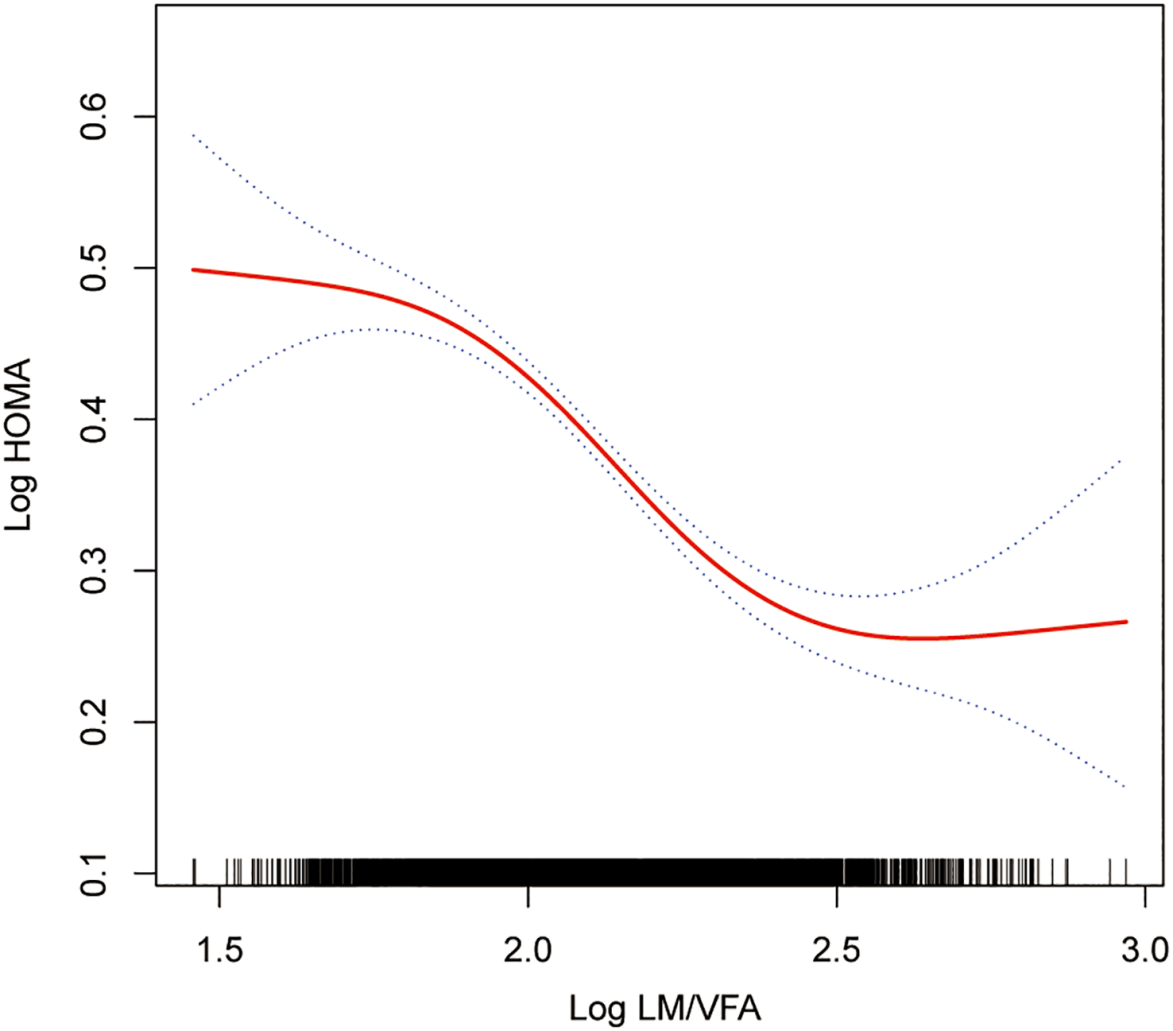
Association between Log LM/VFM and IR. The solid rad line represents the smooth curve fit between variables. Blue bands represent the 95% of confidence interval from the fit. All adjusted covariates were the same as in the regression analysis.

The smoothing curve fitting study revealed an inverse linear relationship between total lean mass and HOMA-IR (**Figure 2**). Visceral fat mass, on the other hand, showed a non-linear upward trend with HOMA-IR (**Figure 3**). Surprisingly, the Log LM/VFM independent exposure variable analysis revealed a substantial negative non-linear relationship with HOMA-IR (**Figure 4**), reaching an inflection point of 1.80 and 2.50, respectively (**Table 3**). A significant inverse correlation was observed between Log LM/VFM and HOMA-IR. The level of HOMA-IR is highest when Log LM/VFM is less than 1.80, and then HOMA-IR decreases as the Log LM/VFM decreases. However, when it exceeds 2.50, the trend becomes flattened. **Supplementary Table 1** describes the characteristics of the variables of interest for populations at different inflection points.

**Table 3:** Threshold effect analysis of Log LM/VFM with HOMA-IR using piece-wise linear regression.

Inflection point of Log LM/VFM	Effect size (β)	95%CI	P-value
<1.8	-0.04	-0.33, 0.24	0.769
1.8˜2.5	-0.35	-0.40, -0.30	<0.001
≥2.5	0.09	-0.13, 0.32	0.415

LM/VFM, total lean mass/visceral fat mass; HOMA, homeostasis model assessment for insulin resistance; β, partial regression coefficient; CI, confidence interval. Adjusted for sex, age, marital status, race, education, family income to poverty ratio, BMI, waist circumference, activity, smoking, alcohol, hypertension, diabetes, total cholesterol, triglyceride, ALT, and blood uric acid.

### 3.3 Subgroup analysis

Subgroup analysis confirmed the association between Log LM/VFM and HOMA-IR with different clinical features. The results of the subgroup analyses based on sex, age, activity level, BMI, hypertension, and diabetes are presented in **Table 4**. Across all subgroups, the studies consistently showed a negative correlation between Log LM/VFM and IR.

**Table 4:** Stratified subgroup analysis between Log LM/VFA and HOMA-IR.

Characteristic	No. of participants	β(95%CI)	*P* -value
Sex			
Male	2397	-0.41 (-0.49, -0.33)	<0.001
Female	2297	-0.33 (-0.39, -0.27)	<0.001
Age			
<30	1461	-0.21 (-0.29, -0.12)	<0.001
30-40	1087	-0.26 (-0.36, -0.17)	<0.001
40-50	1107	-0.31 (-0.41, -0.21)	<0.001
≥50	1039	-0.47 (-0.57, -0.37)	<0.001
Activity			
Active	2551	-0.32 (-0.39, -0.26)	<0.001
Less active	335	-0.28 (-0.46, -0.11)	0.002
Inactive	1798	-0.31 (-0.39, -0.23)	<0.001
BMI			
<25	1599	-0.26 (-0.35, -0.18)	<0.001
25-30	1489	-0.33 (-0.42, -0.24)	<0.001
≥30	1606	-0.29 (-0.37, -0.20)	<0.001
Hypertension			
Yes	1149	-0.28 (-0.38, -0.17)	<0.001
No	3545	-0.31 (-0.36, -0.26)	<0.001
Diabetes			
Yes	549	-0.41 (-0.62, -0.21)	<0.001
No	4145	-0.30 (-0.35, -0.26)	<0.001

LM/VFM, total lean mass/visceral fat mass; HOMA, homeostasis model assessment for insulin resistance; β, partial regression coefficient; CI, confidence interval; BMI, body mass index. Adjusted for sex, age, marital status, race, education, family income to poverty ratio, BMI, waist circumference, activity, smoking, alcohol, hypertension, diabetes, total cholesterol, triglyceride, ALT, and uric acid.

## 4 Discussion

Based on the NHANES survey, the current study revealed a negative correlation between lean body mass and HOMA-IR, whereas visceral fat showed a positive correlation. We used Log LM/VFM as an alternative estimate to understand the combined effect on HOMA-IR. The results showed a negative association between Log LM/VFM and HOMA-IR, with a decrease in HOMA-IR as Log LM/VFM increased. To ensure the credibility of the results, we thoroughly investigated potential confounding factors in the regression analysis to minimize potential bias. Stratified analyses based on sex, age, BMI, exercise level, hypertension, and diabetes were conducted. These findings imply that the conclusion has a high degree of robustness. After additional research on smooth curve fitting and threshold effects, we discovered a nonlinear trend and threshold impact between LogLM/VFM and HOMA-IR.

Researchers have explored the relationship between lean body mass and IR but have yet to reach consistent conclusions. Various studies have demonstrated that increased lean body mass was positively associated with metabolic health. Takamura et al. discovered that lean body mass protects against IR and metabolic abnormalities (30). Similarly, Ghachem et al. found that lean body mass was an independent predictor of IR and that increased muscle mass led to improved IR (31). Ahn et al. investigated IR based on triglyceride glucose index assessment in the Korean population. They found that the group with a low skeletal muscle mass index had higher IR levels (32).

However, other studies have expressed a different view. A study of 7044 participants in the United States showed that lean body mass was positively associated with IR (31). Similarly, a systematic review of children and adolescents showed that lean body mass was minimally associated with glucose homeostasis in children; conflicting views have been generated in the literature (33), and another systematic review had similar findings (34). Rehunen et al. showed that higher lean body mass does not imply freedom from IR. That high lean body mass in men with fatness is detrimental to glucose regulation (35). In addition, a study in community-dwelling older adults in Australia showed that people with sarcopenic obesity did not imply a higher risk of IR (36). Our study supports the hypothesis that lean body mass is conducive to improving IR, and higher lean body mass is associated with lower IR levels, with a significant inverse relationship between the two. The mechanism of action between lean body mass and IR needs to be better understood, and further future exploration of this issue is needed.

The association between adipose tissue and metabolic diseases has received extensive attention. Of particular concern is visceral adipose tissue, which releases cytokines that can circulate throughout the body via the portal vein (37), and deposition of ectopic adipose tissue, contributing to a higher risk of cardiovascular disorders (38). Glintborg et al. found a positive correlation between trunk fat and IR in polycystic ovary syndrome patients (39). Additional studies have also shown a positive association between trunk fat and IR in people with overweight characteristics and a worsening of IR over time (40). The high visceral fat mass has also been positively associated with IR and β-cell dysfunction (41). However, the effects of different body adipose tissues on metabolic health vary. One study in China found that subcutaneous fat reduced the risk of diabetes in women, in contrast to visceral adipose tissue, which increased this risk (42). Given the harmful effects of visceral adipose tissue, we used visceral fat mass as an evaluation indicator to measure its harmful effects on IR better.

Although existing research has separately observed associations between lean mass and adipose tissue with IR, these parts are integral components of the human body that cannot be separated, just like the two sides of a coin. The inconsistent results of previous studies may be partly attributed to inconsistencies in evaluation methods or indices, including using different evaluation methods, such as bioelectrical impedance, dual-energy x-ray absorptiometry, computed tomography scans, and magnetic resonance imaging. Different evaluation indices have also been employed, such as Free Fat Mass, Skeletal Muscle Mass, and Appendicular Skeletal Muscle Index (43). These differences in evaluation methods or indicators limit the consistency between the findings of existing studies. Looking at the correlation in isolation of either lean body mass or adipose tissue with IR can only partially understand the relationship between the variables. Therefore, the combined effect of muscle and adipose tissue concerning IR is worth exploring.

In recent years, researchers have used the biomarker of fat-to-muscle ratio to evaluate body composition. This indicator has been widely used in studies related to various health outcomes, such as mortality (20, 44) and cardiometabolic risk (19). However, the marker of fat-to-muscle ratio was used in previous studies without considering the varying roles of different tissues in the body. Numerous studies have confirmed that visceral fat and subcutaneous adipose tissue were associated with human health-related indicators significantly differently. In a study conducted by Sato et al. (45), it was found that there is a significant association between the visceral fat area and dyslipidemia, hypertension, and coronary atherosclerosis. However, no such association was observed with subcutaneous fat. Huang et al. (46) demonstrated a positive correlation between visceral fat and insulin secretion and sensitivity in a diabetic population, even after considering confounding variables, while subcutaneous fat did not exhibit the same relationship. Similarly, a study by Koenen et al. found that Individuals with visceral tissue deposition are more likely to develop metabolic diseases than similarly obese individuals with less visceral adipose tissue and relatively more subcutaneous fat (47).

The fat-to-muscle ratio has been frequently examined as a metric in previous studies on body composition and insulin sensitivity or IR. A study by Poggiogalle et al. found that individuals with high adipose tissue and low lean mass characteristics tend to have lower insulin sensitivity and higher glycosylated hemoglobin levels (48). Similarly, Habib et al. demonstrated that people with sarcopenic obesity have significantly higher levels of IR and HOMA-β (49). A study conducted by Huang et al. examined 420 patients with type 1 diabetes and found that a higher fat-to-muscle ratio was associated with higher IR and cardiometabolic disorders (50). Another study by Hwang et al. on 424 diabetic patients revealed a positive correlation between the ratio of muscle mass to visceral fat and serum insulin concentrations (51). Based on the results of this study, we can conclude that there is a significant relationship between lean body mass to visceral fat mass ratio and IR. As the ratio increases, the level of IR tends to decrease gradually. However, this relationship is non-linear.

Further analysis of the threshold effect revealed that the level of IR was highest when the log LM/VFM was less than 1.8. Subsequently, as the log LM/VFM decreases, the level of IR also decreases. This finding emphasizes the significance of controlling visceral fat while maintaining lean body mass. The study indicates saturation effect occurs when the log LM/VFM value exceeds 2.5. Beyond this cut-off, increasing lean body mass to visceral fat mass ratio did not significantly improve IR levels. These findings have significant implications for the management of metabolic diseases.

Additionally, we observed variations in the body composition of individuals during different inflection intervals. Participants with a Log LM/VFM>2.5 exhibited significantly lower visceral fat and body weight levels while displaying the highest ratio of lean body mass to visceral fat. On the other hand, those with a Log LM/VFM <1.8 had higher visceral fat and body weight levels and a lower ratio of lean body mass to visceral fat. This higher ratio may be influenced mainly by the lower amount of visceral fat. Whether maintaining a certain level of lean body mass to visceral fat ratio implies a better metabolic health phenotype requires further population-based investigations to verify.

To better understand the research findings, it is vital to acknowledge the limitations of this cross-sectional study. It cannot definitively establish causality, and further cohort studies will be necessary to confirm the results. Secondly, cross-sectional studies are susceptible to confounding variables, which may skew the results. While the researchers attempted to consider these factors, there may still be unknown variables or biases that were not considered during the study that could have led to inaccurate results. Therefore, caution should be exercised when interpreting the findings, and further investigation is required to validate the results under different conditions. Third, in the NHANES survey, only those under 59 completed Dual-energy X-ray absorptiometry, so the study results do not apply to the elderly population. Fourth, diet, water intake, and activity status may influence body composition measurements. We are not sure whether all subjects completed the measures in a fast, peaceful state, which may tend to overestimate body composition results. However, considering all subjects were treated equally and objective measurements were taken at the mobile examination center, this should not bias the estimated results. Fifth, it is essential to note that the results in this study for HOMA-IR show a significantly skewed distribution, so we have log-transformed them. Although this does not change the correlation between the variables, this feature should be noted when interpreting the data. Finally, among the current methods for evaluating IR, the gold standard is the hyperinsulinemic-euglycemic clamp. However, this technique requires special operating equipment, is time-consuming, and is expensive, making it challenging to implement in large-scale population-based surveys. Where possible, in the future, the findings in this study need to be further validated using the gold standard.

In conclusion, it has been observed that there exists a negative correlation between lean body mass and IR. On the other hand, a positive correlation has been found between visceral fat and IR. A nonlinear negative relationship with a threshold effect has been observed through a detailed analysis of the relationship between the lean body mass to visceral fat mass ratio and IR, using LogLM/VFM as a proxy variable.  

## Data availability statement

Publicly available datasets were analyzed in this study. This data can be found here: https://www.cdc.gov/nchs/nhanes/about_nhanes.htm.

## Ethics statement

The studies involving humans were approved by The National Center for Health Statistics Institutional Review Board of the United States. The studies were conducted in accordance with the local legislation and institutional requirements. The participants provided their written informed consent to participate in this study.

## Author contributions

YS and HZ proposed research design. LL and XW cleaned up the data, YH and XZ completed statistical analysis of the data. YS and LL created this manuscript. YS, LL, HZ, XW, YH, and XZ contributed to the discussion, reviewed, and approved the manuscript. LL and HZ made important changes to key elements of the manuscript. All authors contributed to the article and approved the submitted version.
