# Supplementary material for: Anticipated correlation between lean body mass to visceral fat mass ratio and insulin resistance: NHANES 2011-2018
Source: Front Endocrinol (Lausanne). 2023 Sep 13;14:1232896. doi: 10.3389/fendo.2023.1232896 (PMC10526824; doi:10.3389/fendo.2023.1232896)
Supplement: Supplementary file 1 [file Table_1.docx]

**Supplement table S1** Characteristics of participants according to different inflection points.

| Variables | Inflection point | | | |
| --- | --- | --- | --- | --- |
|  | <1.8 | 1.8～2.5 | >2.5 | P-value |
| N | 399 | 4309 | 273 |  |
| Weight (mean ± SD, kg) | 87.55 ± 20.48 | 80.37 ± 20.34 | 65.08 ± 16.75 | <0.001 |
| Lean body mass (mean ± SD, kg) | 50.94 ± 11.82 | 54.38 ± 12.89 | 48.52 ± 12.46 | <0.001 |
| Visceral fat mass (median (IQR), kg) | 0.92 (0.78-1.10) | 0.41 (0.27-0.58) | 0.11 (0.10-0.16) | <0.001 |
| Log LM/VFA | 1.73 ± 0.06 | 2.12 ± 0.18 | 2.60 ± 0.10 | <0.001 |
| Log HOMA | 0.64 ± 0.31 | 0.37 ± 0.35 | 0.13 ± 0.29 | <0.001 |
